# The relationship between drought and tourist arrivals: A case study of Kruger National Park, South Africa

**DOI:** 10.4102/jamba.v9i1.471

**Published:** 2017-07-27

**Authors:** Fhumulani I. Mathivha, Ndivhuwo N. Tshipala, Zanele Nkuna

**Affiliations:** 1 Department of Hydrology and Water Resources, University of Venda, South Africa; 2 Department of Tourism Management, Tshwane University of Technology, South Africa

## Abstract

National parks around the world have been recognised as important sources of nature experiences for both local and international visitors. In South Africa, national parks are similarly important recreational and nature tourism attractions. They offer visitors an unparalleled diversity of tourism opportunities, including game viewing, bush walks and exposure to culture and history. South African National Parks (SANParks), established in 1926, is one of the world’s leading conservation and scientific research bodies and a leading agent in maintaining the country’s indigenous natural environment. The study aims to analyse the correlation between drought and the number of tourist arrivals to the Kruger National Park (KNP). Rainfall data, as well as data on tourist arrivals at KNP for the period from 1963 to 2015 were obtained from the South African Weather Services (SAWS) and SANParks, respectively. Rainfall data were used to determine the drought years at the KNP through computing the Standardised Precipitation Index (SPI) for various stations around the park. Pearson’s correlation coefficient was used as a statistical measure of the strength of a linear relationship between drought and tourist arrivals. The results showed that KNP experienced both negative and positive tourist arrivals, although the former was the case, tourist arrivals showed an increasing trend. The correlation relationship showed that 19.36% of the drought years corresponded to a negative change in tourist arrivals to the park. The results obtained confirm that the tourism industry is a fragile industry which is prone to environmental, social and economic state of a region.

## Introduction

### Problem statement

Climate and weather are some of the important factors taken into account by tourists when deciding on a destination. Weather conditions also influence the successful operation of tourism businesses (Becken 2010). Unfavourable climate and poor weather conditions act as push and pull factors for tourists to travel to warmer, cooler or drier locations (Lise & Tol 2002). Scott and Lemieux (2010) stated that tourism is a significant contributor to national and local economies around the world and is increasingly promoted as having an important role in contributing to the UN Millennium Development Goals (MDGs), particularly the alleviation of poverty in least developed countries. The Kruger National Park (KNP) is one of the largest conservation areas in South Africa and attracts more tourists than any other park in South Africa (Van der Merwe & Saayman 2008). The KNP creates employment in the two main provinces in which the park operates; therefore, if weather conditions are not favourable, the number of tourist arrivals may decline, which would result in unemployment in the Limpopo and Mpumalanga provinces of South Africa. With the growing concern of a changing climate, little attention has been focused towards research that aims to address the economic feasibility of tourism under the changing climate. Southern Africa has observed a warming trend over the past decades, which is consistent with the global trend of temperature rise (Kandji, Verchot & Mackensen 2006). The former creates a favourable environment for the drought phenomenon.

### Study objective

Climate itself is a principal resource for tourism, as it co-determines the suitability of locations for a wide range of tourist activities and is a principal driver of the seasonality of demand (Thomas et al. 2013). Most studies that link climate to tourism have been solely focused on the effects of climate change on tourism. Most tourism studies focus on economic variables (Crouch 1994; Lim, Min & McAleer 2008) as opposed to climate, weather and environment changes. Climate studies, however, have gained momentum over the years as it has been identified as a key driver for tourism and an important destination attribute (Hu & Ritchie 1993). This study, therefore, attempts to evaluate the effects that emanate from drought in the KNP on the arrival of tourists. This will be achieved by correlating Standardised Precipitation Index (SPI) drought years with the percentage of tourist arrivals in the KNP.

## Literature review

### Understanding drought

**FIGURE 1:**
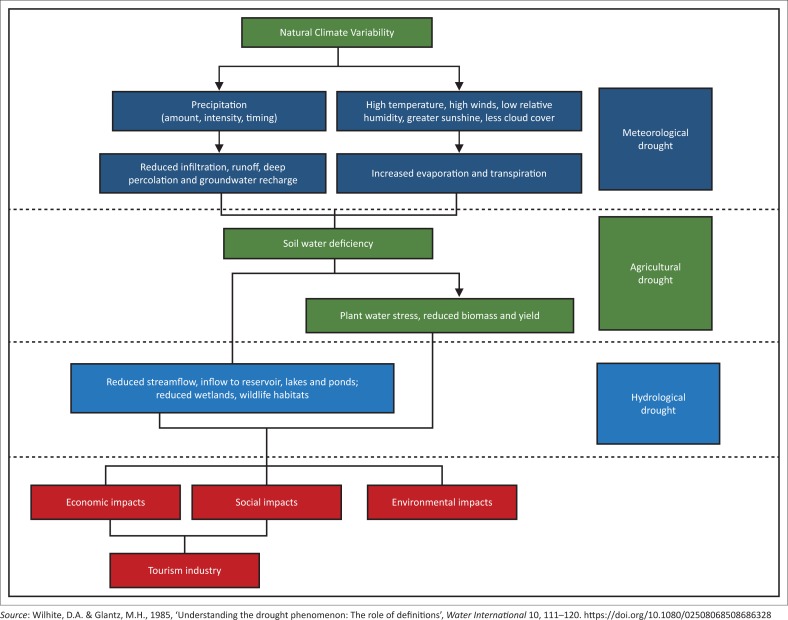
Drought propagation through the hydrological cycle.

Drought is a natural feature of climate. This is a critical natural disaster that adversely affects people, river basins, water resource systems and ecosystems (Jahangir, Sayedur & Saadat 2013). The term ‘drought’ is defined differently in numerous applications (Wambua, Mutua & Raude 2014). However, it is a challenge to quantitatively define the term. Droughts may be expressed in terms of precipitation deficit, soil-water deficit, low stream flows, low reservoir levels and low groundwater level depending on which sector is referred to. The common feature among all sectors is the deficiency in water or moisture. Figure 1 depicts the sequence of drought occurrence through the hydrological cycle and the impacts that emanate thereof. The latter impacts can affect the social aspect (this is communities’ comfort and livelihoods), the environmental aspect (reduced water levels leading to loss of aquatic species, loss of biodiversity) and the economy (poor agricultural returns as a result of poor yields).

There are four main types of droughts as described by Zoljoodi and Didevarasl (2013). The four types of drought are: the hydrological, meteorological, agricultural and socio-economic droughts. The propagation of hydrological and agricultural drought originates from meteorological droughts, which develop from changing phenomena within the hydrological cycle. The lack of precipitation combined with higher evaporation rates propagates through the hydrological cycle from its origin as a meteorological drought into soil moisture depletion to the point where crops or terrestrial ecosystems are impacted, and eventually into a hydrological drought (Mishra & Singh 2010; Tallaksen & Van Lanen 2004; Wilhite 2000).

### Drought and tourism

Tourism is a critical economic sector with mounting evidence linking economic losses to drought, even though the connection is minimally studied or systematically documented (Thomas et al. 2013). In fact, few studies have been conducted relating drought and tourism (Faulkner 2001; Ritchie 2008; Wilhelmi, Hayes & Thomas 2008 in Thomas, Wilhelmi & Hayes 2006). This is particularly relevant as droughts are common in nearly all parts of the globe, with varying frequency, duration and intensity. Their impacts are becoming increasingly complex (Wilhite, Svoboda & Hayes 2007), emerging from a multifaceted interaction between demands for water by humans, animals and the physical availability of water (Hayes, Wilhelmi & Knutson 2004; Wilhelmi & Wilhite 2002).

According to Wilhite and Glantz (1985), the tourism sector is most sensitive to hydrological and socio-economic droughts and is a highly responsive and flexible industry, adapting to demand for new destinations, activities and markets. It is also volatile and sensitive to economic, social, ecological and technological changes. The tourism industry is also particularly vulnerable to the effects of climate change (Becken 2010). Climate change shapes the marketing of many destinations. It shapes tourists’ expectations, experience and memories, which in-turn affect whether people return and where they go next.

Because of its nature of sensitivity, tourism is particularly vulnerable to increases in occurrence of extreme events such as droughts and can at a single destination adversely affect the entire circuit. Drought has direct and indirect impacts on tourism and can span all seasons. The most obvious are reductions in water dependent activities, such as boating, rafting, canoeing and fishing, resulting from lower water levels, as well as from shortened or shifted seasons (Ding, Hayes & Widhalm 2011). Water restrictions can also pose distinct challenges to water-reliant recreation. Intangible relationships are more difficult to quantify and link to drought, such as decreased visitation, cancellations of hotel stays or a reduction in booked holidays (Schneckenburger & Aukerman 2002). A healthy tourism sector is vital for overall social and economic strength in communities and regions, particularly in places where tourism makes up a significant portion of the overall economy. Consequently, systematic and consistent assessments and evaluation of drought impacts, along with direct inclusion in drought management strategies are fundamentally necessary, although thus far not commonly conducted for tourism (Wilhelmi et al. 2008). Currently, the KNP has many strategies including good working relationship with its upstream neighbours, which means that even during dry times, water is being let through to serve the KNP at the bottom end of the river catchments (Van Vuuren 2016).

Tourism and recreation activities do not take place in a vacuum and as such interact with many other sectors. As has already been mentioned, many of the outdoor recreation activities are dependent on the availability of water in large quantities. As the area becomes drier and experiences more drought conditions, recreational water users will be competing with farmers and industry for that water resource (Wall 1998). This holds the potential for conflicts among users for limited supplies of good quality water. Other resource conflicts that can arise are, for example, between recreationists and forestry, mining and commercial fishing industries (Wittrock, Baird & Wheaton 1992; Wittrock & Wheaton 1992).

## The Kruger National Park

**FIGURE 2:**
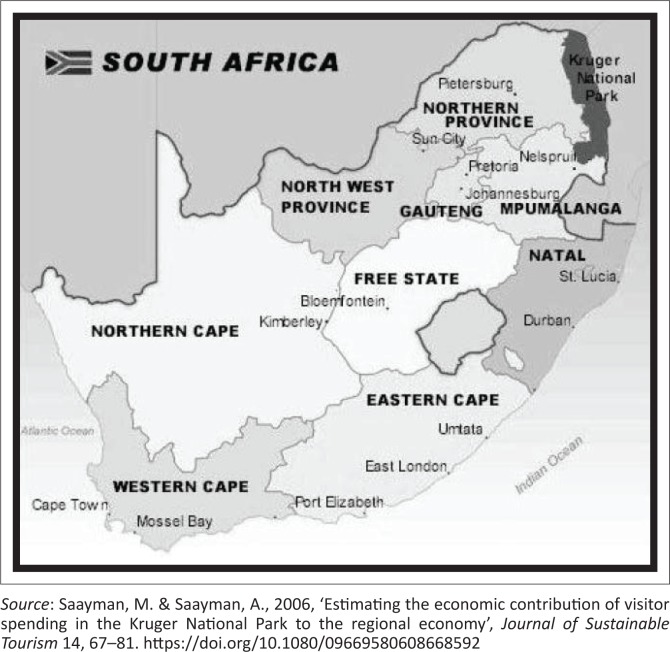
Kruger National Park.

The Kruger National Park was established in 1927. Over the past decades, the park has grown both in terms of infrastructure and in the number of tourists it attracts each year. The park is situated in the north-eastern part of South Africa (23.9884° S, 31.5547° E) and is one of Africa’s largest game reserves. It covers an area of 19 485 km^2^, as shown in Figure 2. The climate of the KNP is mostly semi-arid with mean annual temperature and precipitation of 22 °C and 550 mm/annum, respectively, and an average potential evaporation of 7 mm/day (Du Toit, Rogers & Biggs 2003). Its high density of game includes the Big 5: lions, leopards, rhinos, elephants and buffalos. Hundreds of other mammals make their home here, as do diverse bird species such as vultures, eagles and storks. Mountains, bush plains and tropical forests are all part of the landscape within the park.

South African National Parks (SANParks) does not only contribute to South Africa’s nature conservation but it also has a positive influence on the country’s economy. The tourism industry consists of a number of different but interlinked service industries, particularly accommodation and catering, food processing and beverages, transport, as well as entertainment and other support services (Paton 1985). The KNP is the single largest and most significant tourism product in the Limpopo and Mpumalanga provinces and spending by visitors within the park represents a 5.72% estimate of the total value of tourism spending in the province. The total number of people directly employed in the tourism industry in Limpopo is 32 888, excluding the KNP (Saayman, Slabbert & Saayman 2002). Together with the KNP, this amounts to 7.21% of the total employment in the Mpumalanga province, which is higher than the employment in the electricity, construction and finance industries in the province. If the spending information from the KNP is compared to that of other South African Parks (Saayman et al. 2002), the KNP exceeds that of any other park, as does the availability of various products. Therefore, from a park development point of view, it is very important to create an environment for tourists to spend. This will also contribute to the economic sustainability of the KNP.

## Methodology

### Data requirements

Rainfall data for a period of 30 years (1980–2010) and tourist statistic data over a period of 52 years (1963–2015) were obtained from SANParks. Rainfall data were used to determine the drought years in the KNP while tourist data (total number of tourists, international tourists and local tourist) were used to determine the frequency of both local and international visitors to the KNP. The choice of 30 years rainfall was based on the fact that this study is focused on assessing the impacts of long-term change of the climate on the tourism industry. The World Meteorological Organization (WMO) recommended a study period of not less than 30 years as ideal, while studying long-term climate change. Drought years were determined using the SPI for five stations (Letaba, Shingwedzi, Skukuza, Satara and Phunda Maria) within the KNP. SPI has been widely used in drought assessment and monitoring (Rouault & Richard 2003; Türkeş & Tatl 2009; Vicente-Serrano 2006; Wu et al. 2005).

### Determination of drought years

The Standardised Precipitation Index was used to quantify rainfall deficit within the KNP as a representative of drought condition. The standardisation procedure transforms rainfall data to come up with standardised anomalies. The advantage of using the standardisation procedure is that it aids in discerning normal and typical values and is symmetrical for the occurrence of wet and dry events (Sutton & Kempi 1993). The data were standardised using the formula as defined by Goddard and Melville (1996) in Equation 1:

$$Z=\frac{X_{i}-\bar{x}}{\sigma }$$ [Eqn 1]

where $\bar{x}$ = sample mean, *Z* = normalised standardised departure, *x_i_* = raw value and σ = sample standard deviation. Table 1 describes values of the SPI with corresponding appropriate classification of the severity by McKee, Doesken and Kleist (1995). An SPI value of 2.0 or greater is an indicator of extreme wet conditions (flood), while an SPI value of −2.0 or less is an indication of extremely dry conditions (drought).

**TABLE 1:** Categories of the Standardised Precipitation Index based on the Standardised Precipitation Index value.

Category	SPI
Extremely wet	< 2.0
Severely wet	1.5 to 1.99
Moderately wet	1.00 to 1.49
Near normal	0 to −0.99
Moderate drought	−1.00 to −1.49
Severe drought	−1.5 to −1.99
Extreme drought	< −2.0

SPI, Standardised Precipitation Index.

### Determination of correlation between drought and tourist arrivals

This study employed statistical correlation of climatic and the tourist arrival data to achieve the objective of the study. The linear relationship between two continuous quantities is often assessed in terms of Pearson’s Correlation Coefficient (PCC) (Marzban et al. 2013). PCC is a statistical measure of the strength of a linear relationship between two variables and is denoted by *r*. A value of 0 denotes no linear correlation, while a value closer to 1 or −1 resembles a stronger positive or negative linear correlation. The PCC for this study will be determined using Equation 2:

$$r=\frac{n\sum xy-\sum x\sum y}{\sqrt{\left(n\sum x^{2}-{\left(\sum x\right)}^{2}\right)-\left(n\sum y^{2}-{\left(\sum y\right)}^{2}\right)}}$$ [Eqn 2]

where *n* is the number of occurrences, *x* is the tourist population and *y* is the drought years. The *r* was then converted to *R*^2^ for further analysis of the correlation between the change in tourist arrivals and the computed SPI.

## Results

### Tourist arrivals at Kruger National Park between 1963 and 2015

**FIGURE 3:**
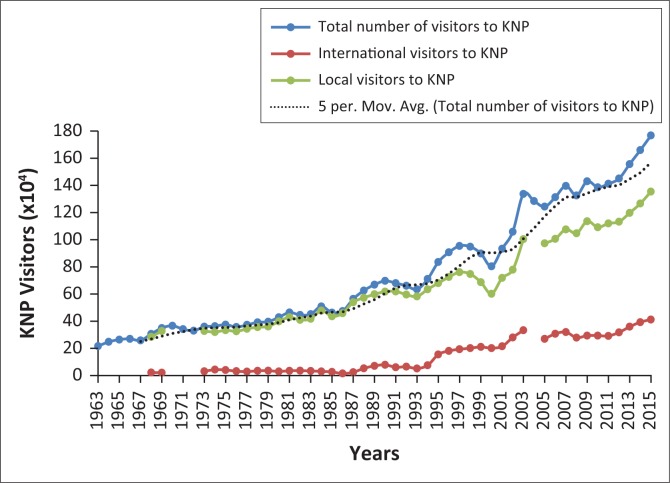
Visitors to Kruger National Park.

Figure 3 illustrates the state of KNP tourist arrivals over 52 years (1963–2015). The analysis depicts the number of both domestic and international visitors. It is evident from the figure that more South Africans are visiting the park than international visitors; however, the local and international visitors are showing an increasing trend under the period considered in this study. A 5-year moving average was fitted to the total number of tourist arrival data (as can be seen in Figure 3). This was done to highlight any significant changes in the tourist arrival trends to the KNP. It also provided valuable insights into the trends of the past and presents a foundation for predicting future trends. The fitted 5-year moving average trendline shows an exponentially increasing trend of tourist arrivals within the park.

Over the years the KNP has experienced both positive and negative changes in terms of the number of visitors received year on year. The negative and positive changes can be either as a result of social change, park infrastructure development, or ecological and environmental changes. Buckley and Klemm (1993) indicated that some factors that may affect tourism in a region include, but are not limited to, administrative structures of a region, terrorism, weather conditions and civil unrest. A positive change was 18.3% in the 1968/1969 financial year; this was attributed to the Lower Sabie Camp remaining open for the first time during the summer months. In the 2000/2001 financial year, the park witnessed a negative setback of 10.5% change in the number of visitors who visited the park. This was probably as a result of major floods that struck southern Africa in the year 2000 and forced many camps to be closed for extended period of time.

### Analysis of drought years in the Kruger National Park

**FIGURE 4:**
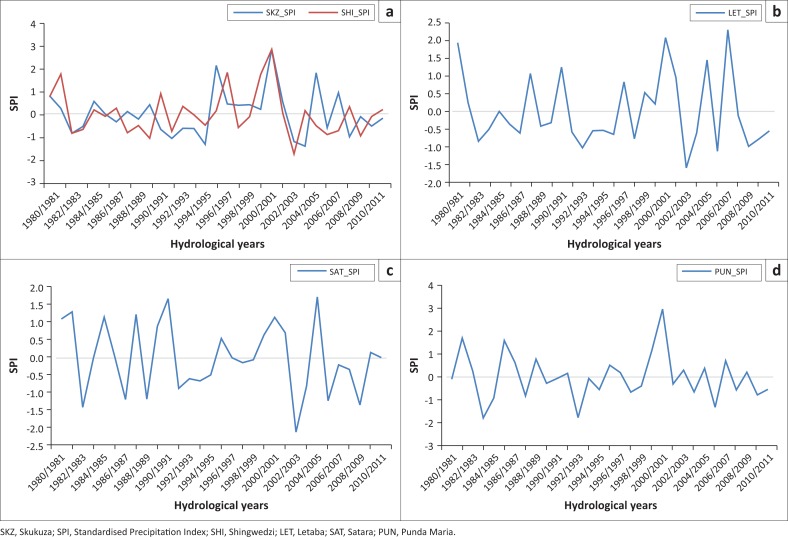
Kruger National Park Standardised Precipitation Index. (a) Skukuza and Shingwedzi Standardised Precipitation Index, (b) Letaba Standardised Precipitation Index, (c) Satara Standardised Precipitation Index and (d) Punda Maria Standardised Precipitation Index.

The analysis of drought year’s frequency for this study was achieved through the standardised procedure as outlined in this section. Figure 4 is the computed SPI for the Letaba (LET_SPI), Shingwedzi (SHI_SPI), Skukuza (SKZ_SPI), Satara (SAT_SPI) and Phunda Maria (PUN_SPI) rainfall stations. The figure shows the range of SPI values in the study area ranging from −3 to +4 (SHI_SPI and SKZ_SPI), −2 to +2.5 (LET_SPI), −2.5 to +2 (LET_SPI) and −3 to +4 (PUN_SPI).

Stations SHI, SKZ and PUN depicted 17 drought years, LET depicted 19 drought years, while SAT depicted 15 drought years of the 31 years recorded. That is 54.84%, 61.29% and 48.39% of drought occurrence in the respective stations in the KNP, respectively. The latter is an indication that drought is more prevalent in the region. Throughout the 20th century, droughts have occurred all over South Africa with great regularity (Vogel 1995). The lowest SPI for LET, SHI, SKZ, PUN and SAT was −1.57, −1.79, −1.44, −1.79 and −2.10, respectively. The latter was experienced in 1983/1984 (PUN), 2002/2003 (LET, SHI and SAT) and 2003/2004 (SKZ) hydrological years.

Mason and Tyson (2000) indicated that major drought years in South Africa occurred in 1991/1992, 1997/1998 and 2001/2002 hydrological years. The study by Mason and Tyson (2000) is in agreement with the results of this study as the major dry year computed was in 1991/1992. Glantz, Betsill and Crandall (1997) and Vogel, Laing and Monnik (2000) reported that the 1991/92 drought as one of the worst recent droughts on record in the country because of the far-reaching impacts felt through all sectors of society. Other significant drought years that were observed from the analysis included the 1982/1983, 1983/1984, 1993/1994, 2004/2005 and 2011/2012 hydrological years. FAO (2004) reported four major droughts in the Southern Africa Development Community, notably the 1982/1983, 1987/1988, 1991/1992 and 1993/1994 hydrological years. Although the major drought (lowest SPI value) for the selected stations is not falling within the major drought years reported by FAO (2004), Glantz et al. (1997), Mason and Tyson (2000) and Vogel et al. (2000), the KNP still experienced drought within those major drought years (see Table 2). Of the eight major droughts reported in southern Africa, seven were experienced in the SHI station of the KNP, while SKZ and SAT experienced four and PUN and LET experienced five drought years each.

**TABLE 2:** Major drought comparison.

Drought years	SKZ	SHI	SAT	PUN	LET
1982/1983	✔	✔	✔	-	✔
1983/1984	✔	✔	-	✔	✔
1987/1988	-	✔	-	✔	-
1991/1992	✔	✔	✔	-	✔
1993/1994	✔	✔	✔	✔	✔
1997/1998	-	✔	✔	✔	✔
2001/2002	-	-	-	✔	-
2004/2005	-	✔	-	-	-

SKZ, Skukuza; SHI, Shingwedzi; SAT, Satara; PUN, Punda Maria; LET, Letaba.

### Correlation between tourist arrivals and the computed Standardised Precipitation Index

**FIGURE 5:**
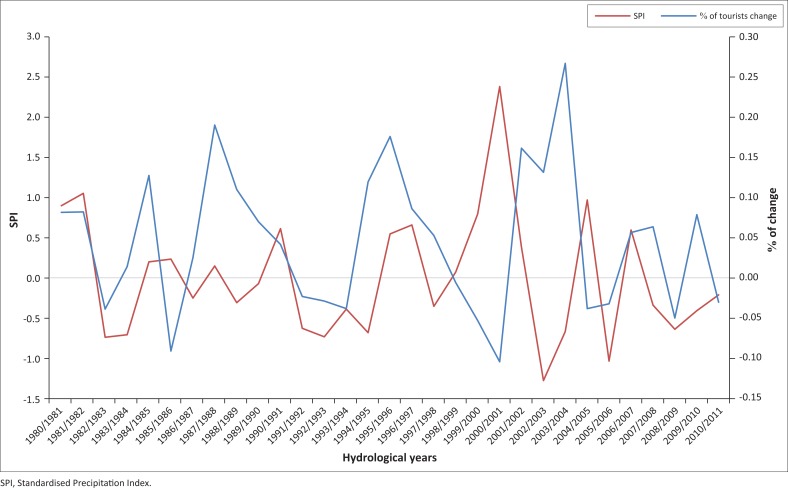
Correlation of percentage of tourist change to the Kruger National Park and the computed Standardised Precipitation Index.

Figure 5 illustrates the correlation between the percentage of change of tourists to KNP and the drought years given by the computed SPI. This can be noted from the figure evidently the year to year fluctuations on both the computed SPI and the percentage of tourist change. From the analysis, the KNP experience negative changes in 1981/1982, 1984/1985, 1990/1991, 1991/1992, 1992/1993, 1997/1998, 1998/1999, 1999/2000, 2003/2004, 2004/2005, 2007/2008 and 2009/2010. 1982/1983, 1983/1984, 1986/1987, 1988/1989, 1989/1990, 1991/1992, 1992/1993, 1993/1994, 1994/1995, 1997/1998, 2002/2003, 2003/2004, 2005/2006, 2007/2008, 2008/2009, 2009/2010 and 2010/2011 were identified as drought years in the KNP as per the five stations’ mean-computed SPI.

Of the 31 years considered in the study, 5 years (19.36%) of the drought years corresponded to the percentage of negative change of tourist arrivals in the KNP. The coefficient of determination (*R*^2^; the squared correlation coefficient) was found to be 0.02. *R*^2^ ranges from 0 to 1, with higher values indicating less error variance, and typically values greater than 0.5 are considered acceptable (Santhi et al. 2001; Van Liew, Arnold & Garbrecht 2003). Therefore, the *R*^2^ obtained in this study is low (< 0.5) indicating an insignificant statistical correlation between the SPI and the percentage of change in tourist arrivals to the KNP. The correlation results obtained in this study are in agreement with that of Vido et al. (2016). The former study obtained varying correlation (0.1, 0.05, 0.06 and 0.01) for different SPI time scales while correlating SPI with physiological characteristics.

Although a low correlation is observed between drought years and tourist arrivals, there are some drought years that are correlating to the negative change in tourist arrivals over the park. For instance, the steep decline in the number of visitors in 1984/1985 may not only be attributed to the social unrest the country experienced during that period, but also there were two consecutive droughts (1982/1983 and 1983/1984) prior to the unrest and could also have contributed to the decrease in the number of tourist arrivals to the KNP. South Africa hosted the Soccer World Cup in 2010, and as a result, the country expected to have a high number of international tourists in the entire country (FIFA.com 2010). This, however, was not the case for the KNP, as the park reported a negative change in terms of the percentage of tourist arrivals in 2010/2011. Similar to the case of the decline in 1984/1985, there were two consecutive droughts prior to 2010 (2007/2008 and 2008/2009), while 2010 was also classified as a drought year in the park as per the computed SPI. Drought has also been identified as one of the natural disasters apart from floods that negatively impact the tourism industry (South African Weather Service [SAWS], 2008 in Shaw, Saayman & Saayman 2012). Therefore, it is further emphasised that the tourism industry is a fragile industry similar to any other industry and is prone to changes in environmental, social and economic states of a country.

## Conclusion

This study determined the relationship between tourist arrivals and drought in the KNP in north-eastern part of South Africa. Historical rainfall data from SAWS were used to determine drought years in the park, while historical tourist arrival data obtained from SANParks were used to determine the frequency of tourist arrivals to the park. The results showed that KNP experienced both negative and positive tourist arrivals, although the former was the case, tourist arrivals showed an increasing trend. The drought years obtained in this study were in agreement with those reported by FAO (2004), Glantz et al. (1997), Mason and Tyson (2000) and Vogel et al. (2000) in the region the study area is located. The correlation between drought and tourist arrivals in the KNP showed that 19.36% of the drought years corresponded to negative change of tourist arrivals in the park. Although a low correlation is observed, there are years in the negative change that coincided with drought years experienced within the park. South Africa hosted the soccer world cup in 2010, and as a result, the country expected to have a high number of tourists in the entire country. The former, however, was not the case for the KNP as the park reported a negative change in terms of the percentage of tourist arrivals in 2010/2011. Therefore, this study concludes that drought is one of the natural disasters apart from floods that negatively impact the tourism industry.
